# Validation of the contralateral side as reference for selecting radial head implant sizes

**DOI:** 10.1007/s00276-016-1625-x

**Published:** 2016-01-21

**Authors:** Paul W. L. Ten Berg, Johannes G. G. Dobbe, Gerhard van Wolfswinkel, Simon D. Strackee, Geert J. Streekstra

**Affiliations:** 1 Department of Plastic, Reconstructive, and Hand Surgery, Academic Medical Center, University of Amsterdam, Room G4-226, Meibergdreef 9, 1105 AZ Amsterdam, The Netherlands; 2 Department of Biomedical Engineering and Physics, Academic Medical Center, University of Amsterdam, Amsterdam, The Netherlands; 3 Department of Orthopaedics, Academic Medical Center, University of Amsterdam, Amsterdam, The Netherlands; 4 Department of Radiology, Academic Medical Center, University of Amsterdam, Amsterdam, The Netherlands

**Keywords:** Radial head arthroplasty, Radial head implant, Radial head fracture, Radial head, Proximal radius

## Abstract

**Purpose:**

In arthroplasty of comminuted radial head fractures, the contralateral radial head diameter can be used as reference for implant selection. However, potential bilateral asymmetry may result in a mismatch of the implant with the native bone. Therefore, our purpose was to evaluate anatomical right-to-left differences of radial head diameters. We also compared conventional two-dimensional (2D) with three-dimensional (3D) measurements.

**Methods:**

We used bilateral CT-scans from 25 intact proximal radius pairs of right-handed adult subjects to obtain 50 3D radial head models. After contralateral matching, diameters were calculated using a 3D-based method using an automated circle-fit in standardized cross-sections at the widest level midway through the radial head. The 3D-based diameters were compared to orthogonal line measurements in standard axial CT-slices.

**Results:**

Three-dimensional analysis yielded a radial head diameter of 23.0 ± 1.7 mm. The dominant right side was significantly wider, with right-to-left differences of 0.2 ± 0.4 mm, with a maximum of 0.9 mm. The 2D-based diameter was 22.9 ± 1.7 mm, which was 0.1 ± 0.3 mm smaller compared to corresponding 3D-based diameter.

**Conclusions:**

In healthy radial heads, the diameter was biased to the dominant right side, but individual differences were not larger than 1 mm. Compared to implant designs, in which diameter increments are usually 2 mm, this right-bias is not clinically relevant, as it would not affect implant selection. Therefore, the contralateral side can be considered a suitable reference. In clinical practice, the surgeon could estimate this diameter using standard axial CT slices, since its difference with the 3D-based evaluation was also relatively small compared to implant sizing increments.

## Introduction

Radial head arthroplasty is a well-accepted procedure in the treatment of comminuted unreconstructable fractures or post-traumatic arthritis [1]. The implant should approximate the size of the native radial head to replicate the native joint kinematics and avoid postoperative pain, decreased range of motion, and eventually osteoarthritis of the capitulum [2, 3]. Adequate implant sizing is therefore an important aspect of arthroplasty.

Recent research focused on methods for predicting the native diameter of injured radial heads, as reference for implant selection [4–6]. Alolabi et al. [5] stated that the excised radial head, when available, should be used to select the implant. However, assessing the native bone shape becomes more difficult in cases of a high degree of comminution, or open fractures with missing bone. In these injuries, the surgeon can use the opposite healthy bone as reference to estimate the native head diameter. A prerequisite for this approach is the existence of sufficient bilateral anatomical symmetry. One cadaveric study showed that left-sided radial heads are similar to right-sided heads [7]. However, this study was limited by having a relatively small sample size (eight radial head pairs), and not taking dominance into account. Dominance is an important aspect in anatomic studies. One study, for example, analyzed bilateral symmetry of the radius, and showed that the dominant right side was generally longer [8].

The main purpose of this anatomic imaging study was to investigate the bilateral symmetry of normal radial head pairs obtained from 20 healthy right-handed volunteers. To this end, we used three-dimensional (3D) computed tomography (CT) analysis, providing detailed 3D information of bony anatomy, using standardized measurements [9–11]. We quantified right-to-left differences of the outer diameter of the paired 3D radial head models, and hypothesized that there was no bias between right and left. Our second purpose was to evaluate to what extent conventional measurements in standard axial CT slices, comparable with common practice, are in agreement with the aforementioned 3D-based measurements of the radial head.

## Materials and methods

### Data acquisition

In this study we used bilateral CT-scans of intact forearms including the proximal radius obtained from a historical group available from previously conducted experiments [8]. This group included 20 healthy right-handed volunteers (14 women and 6 men; average age 28 years; range 22–56 years). The volunteers had no history of elbow injury or other musculoskeletal disorders. The volunteers confirmed to be right-handed. To increase the number of male subjects, we added bilateral forearm CT scans from five male patients (average age 31 years; range 18–45 years) which were treated for a unilateral distal or midshaft radius malunion. The patients had also no history of elbow injury. For all proximal radii pathology was ruled out based on radiological reports and on reviewing the images again. All patients also confirmed to be right-handed. High-resolution bilateral CT scans (Philips Brilliance 64 CT scanner, Amsterdam, The Netherlands) were made using standardized methods (voxel size 0.45 × 0.45 × 0.45 mm, 120 kV, 150 mAs, pitch 0.6, Slice thickness 0.67 mm). This study was approved by our Human Research Committee. Informed consent of each volunteer was obtained prior to participation.

## 3D bone modeling

**Fig. 1:**
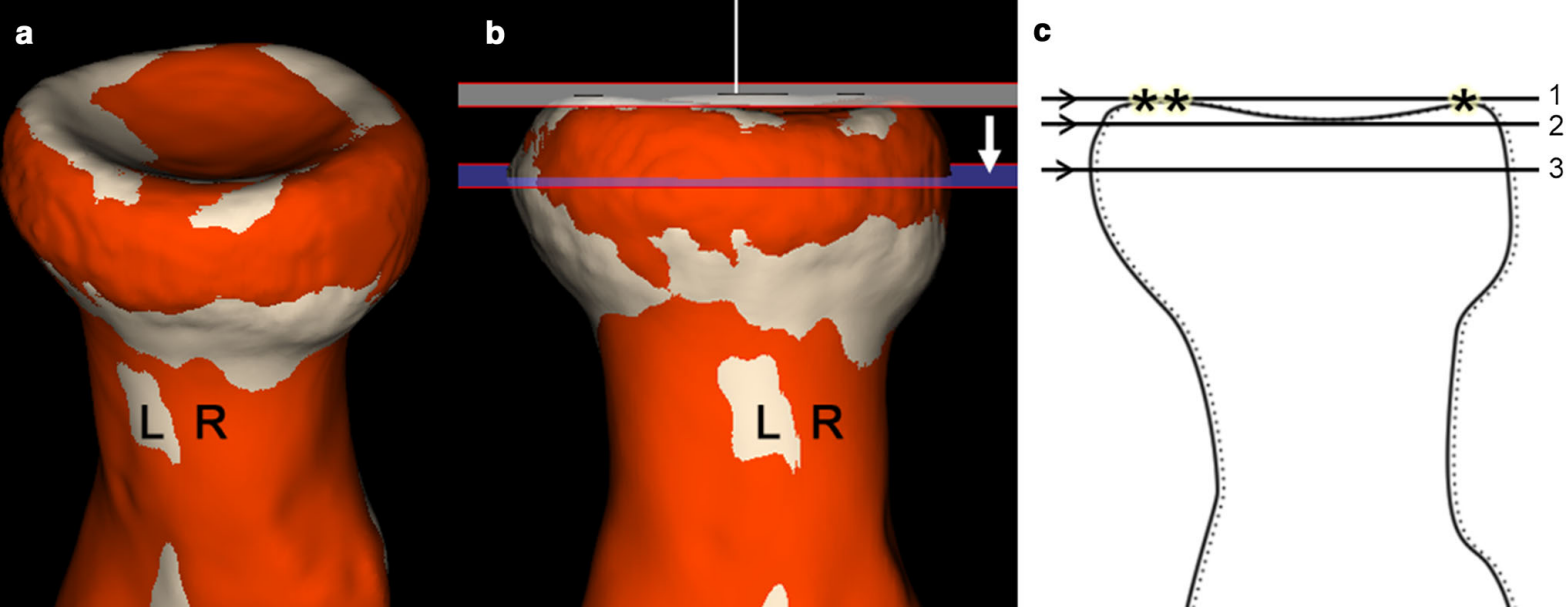
**a** Aligned 3D models of right (*R*) and left (*L*) proximal radii. **b** 3D models of the proximal *R* and *L* radii with a cross-sectional plane. This plane was positioned at the widest level parallel to a reference plane fitted tangentially to the three most proximal points (*asterisk symbol*) on the proximal radial head surface. **c** Scheme of the proximal *L* and *R* radii with a reference plane (*1*) and the parallel cross-sectional planes to cross-section the radius head at the level of the trough (*2*), and at the widest level (*3*) of the radial head

Twenty-five bilateral CT-scans were used to obtain 40 virtual 3D models of left and mirrored right radii based on custom made 3D image segmentation software [12]. Next, we selected the proximal radial head of the left side, and matched each to the opposite side in a semi-automated fashion based on image registration (Fig. 1a) [12, 13]. This matching enabled visual inspection of shape symmetry. It further enables selecting the same level for the cross-sectional diameter calculation of right and left radius models.

## 3D-based determination of the radial head diameter

**Fig. 2:**
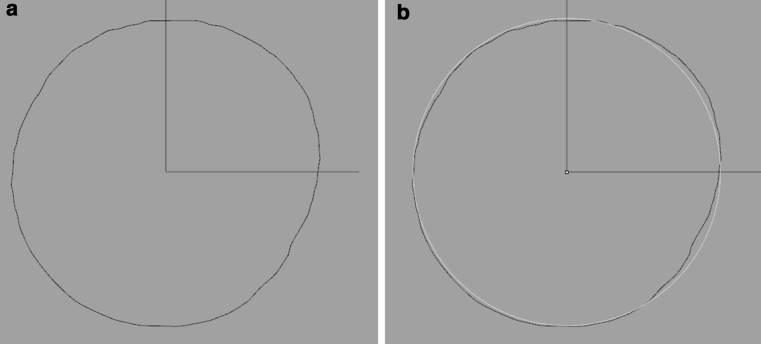
**a** A 2D contour of a radial head obtained after cross-sectioning the 3D model at the widest level. **b** An automated 2D circle fit (*yellow circle*) through the contour enabling calculating the outer diameter of the radial head

A plane was used to cross-sect the widest region of the radial head and to subsequently determine the outer diameter. Since the right and left radial head models were aligned by registration, only one plane was sufficient to cross-sect both models from each volunteer. The 3D orientation of this plane was set in a standardized fashion. First, we fitted automatically an axial reference plane onto the most proximal part of the left radial head using three tangential points based on a custom written algorithm (Fig. 1b, c). A plane parallel to this reference plane was manually shifted to the widest point midway through the radial head. By cross-secting the paired radial head models, right and left 2D contours of the cortical bone were obtained (Fig. 2a). Next, we fitted automatically a 2D circle through each contour using a least-squares circle fit (Fig. 2b). The diameter of this best-fitting circle served as measure of the outer diameter of the radial head.

As additional measure, we used a second plane parallel to the reference plane to cross-sect the radial head at a more proximal level of the trough of the radial head (Fig. 1c) [14]. A similar best-fitting circle was used to obtain a diameter at this level. The 3D-based measurements were independently performed by two observers (research fellows; P. W. B and G. W).

## 2D-based determination of the radial head diameter

**Fig. 3:**
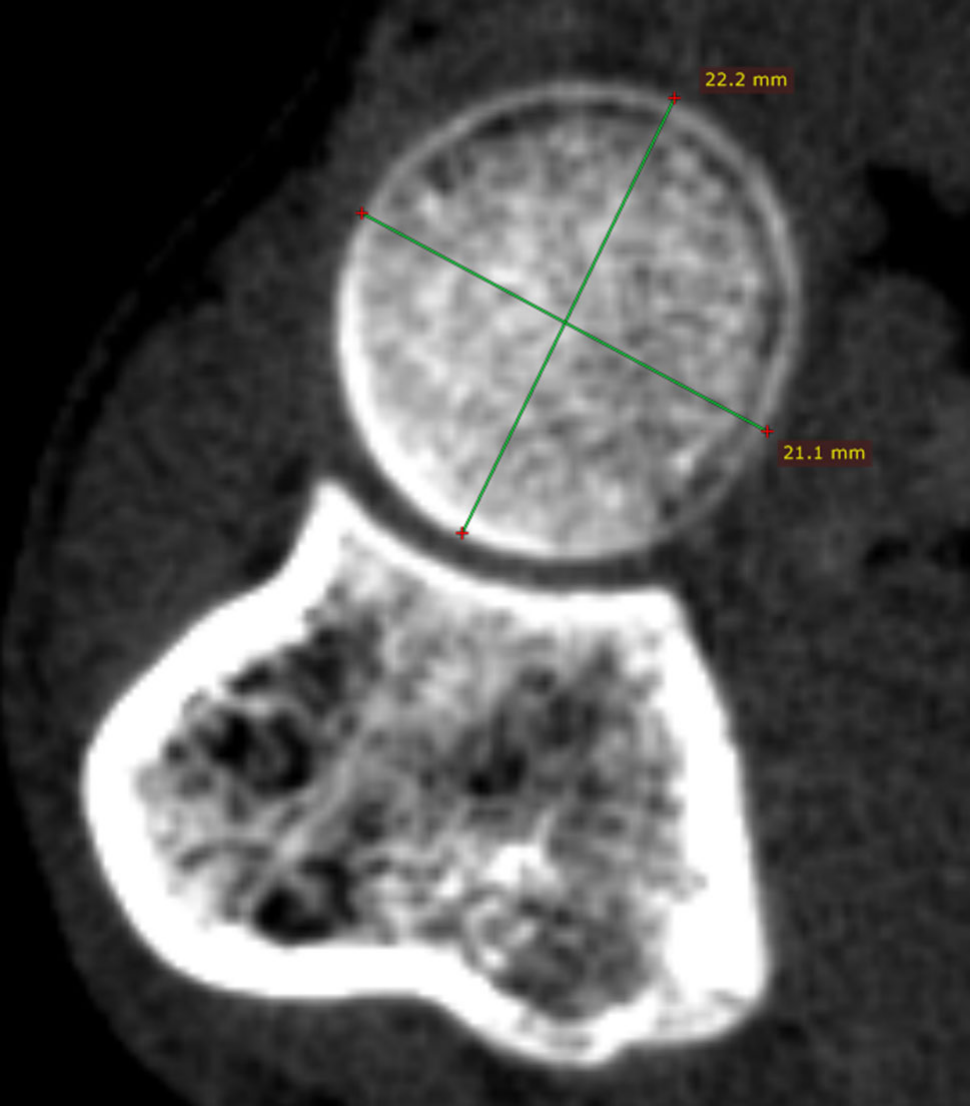
Axial CT slice of the radial head at the widest level. The minimum and maximum diameters were visually selected by drawing two different orthogonal lines. The average 2D-based diameter was based on the average of the minimum and maximum diameter

In the second phase, we measured the on-screen radial head diameter interactively at the widest level using orthogonal line measurements in standard axial CT slices, and compared them with the corresponding 3D-based diameters. First, the slice showing the widest part of the radial head was chosen. In this slice, we visually selected the minimum and maximum length (i.e., minimal and maximum 2D-based diameter) [14], and calculated the average of these two, which served as average 2-D based diameter (Fig. 3). The 2D-based measurements were independently performed by two observers (research fellows; P. W. B and G. W).

### Statistical analysis

Statistical analyses of the measurements included the Shapiro Wilks *W* test as normality test, and determining the mean, and standard deviation (SD) for normally distributed data. The correlations between diameters of right and left sides and a best-fitting line through the points were calculated using linear regression modelling. Right-to-left differences were calculated by subtracting the 3-D based diameter obtained from the left side from the diameter obtained from the right side (right minus left diameter). A one-sample Student *t* test was used to test if right-to-left differences were significantly different from zero, indicating a left- or right-bias. A post hoc power analysis for one-sample *t*-test was used to calculate what mean right-to-left differences of diameters at the widest level could have been tested on significance with sufficient power. This power analysis requires input of the sample size (*N* = 25), comparison mean (=0), and standard deviation, while using an α-level of 0.05 and a power of 0.80.

Regarding the 2D-based diameters, minimum and maximum diameters were compared with a paired Student *t* test. Next, for each radius, we calculated the differences between the average 2D-based diameter and the corresponding 3D-based diameters (3D-based minus 2D-based diameter).

Interobserver agreement was calculated using the intraclass correlation coefficient (ICC) through a two-way mixed effects model with absolute agreement [15]. An ICC above 0.8 indicates very high interobserver agreement. A 5 % significance level was used for all analyses.

## Results

**Fig. 4:**
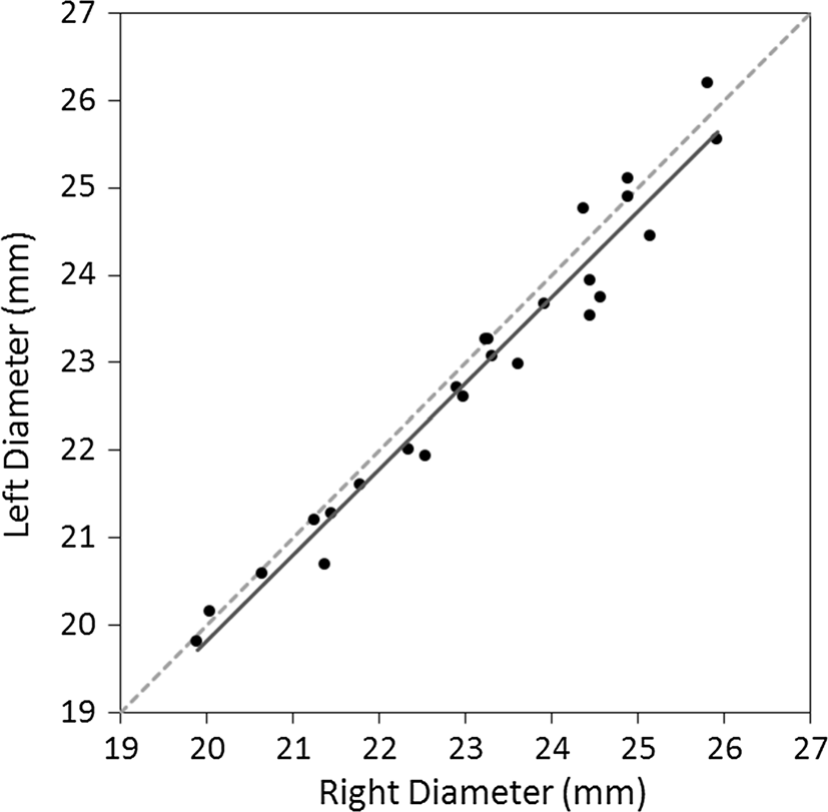
Graph showing individual right- versus left-sided radial head diameters at the widest level (*r* = 0.98) obtained by 3D-based diameter evaluation. The best-fitting line (*solid black line*) through the points deviated from the diagonal line of identity (*dashed grey line*), indicating slightly larger diameters for the dominant right side

In this section all anatomical measurements are expressed as mean ± standard deviation (SD) unless stated otherwise. All evaluation parameters were normally distributed. Based on the total sample, the 3D-based diameter at the widest level of the radial head was 23.0 ± 1.7 mm (males 24.5 ± 0.9 mm; females 21.9 ± 1.2 mm). The right–left correlation coefficient of radial head diameters at the widest level was very strong [*r* = 0.98 (*p* < 0.001)]. The best-fitting line through the points ran parallel to the line of identity (right diameter = left diameter), but with a slight bias towards the right side indicating that the dominant right side was generally wider (Fig. 4). The right-to-left difference of the diameter was 0.24 ± 0.4 mm, with a maximum of 0.9 mm (Table 1). Based on this latter standard deviation, there was sufficient power to detect a significant right–left bias if the mean difference was >0.18 mm. Our reported mean was larger than this cut-off, and statistically different from zero (*p* = 0.003). This confirmed that, in our sample, right-to-left differences were biased to the right side. Regarding anatomical measurements at level of the trough, the 3D-based diameter was slightly, but consistently smaller (22.6 ± 1.7 mm), with comparable right-to-left differences (0.31 ± 0.4 mm) which were also significantly different from zero (*p* = 0.001). A subgroup analysis including only the healthy volunteer data did not alter significance of right-to-left differences. The ICC between the two observers for the diameters as measured at the widest level and at level of the trough were both 0.99, indicating very high interobserver agreement.

**Table 1:** Right and left radial head diameters measured using 3D-based evaluation at the widest level in intact proximal radius pairs (*N* = 25)

Side	Diameter	Difference (R minus L)	
	Mean ± SD	Mean ± SD	|maximum|*
Right (*R*)	23.2 ± 1.7		
Left (*L*)	22.9 ± 1.7	0.2 ± 0.4^†^	0.9

* Absolute maximum

^†^Significantly different from zero (*p* = 0.003)

Regarding the 2D-based diameters, the minimum diameter was 22.2 ± 1.5 mm, the maximum diameter 23.6 ± 1.8 mm (difference: *p* < 0.001), and the average diameter 22.9 ± 1.7 mm. The ICC between the two observers for the average diameter was also 0.99, indicating very high interobserver agreement. The average 2D-based diameter was significantly smaller than the corresponding 3D-based diameter (*p* = 0.013), with a difference of 0.1 ± 0.3 mm, with a maximum of 1.0 mm (Table 2).

**Table 2:** Radial head diameters measured using on 3D-based and 2D-based evaluation at the widest level in intact proximal radii (*N* = 50)

Evaluation type	Diameter	Difference (3D minus 2D)	
	Mean ± SD	Mean ± SD	|maximum|*
3D-based	23.0 ± 1.7		
2D-based (minimum)	22.2 ± 1.5	0.8 ± 0.5^†^	2.0
2D-based (maximum)	23.6 ± 1.8	−0.5 ± 0.4^†^	1.8
2D-based (average)	22.9 ± 1.7	0.1 ± 0.3^†^	1.0

* Absolute maximum

^†^Significantly different from zero (all *p* < 0.01)

## Discussion

In arthroplasty of the radial head, most radial head implant designs are circular and come in fixed ratios of height and diameter. They usually change with diameter increments of 2 mm [4, 16]. An important aspect in choosing implant size is estimating the native radial head diameter [4]. When using the contralateral side as reference, presence of sufficient anatomical bilateral symmetry is a prerequisite. Potential right-to-left differences may result in a mismatch between the implant and the native anatomy. In this article we evaluated whether the contralateral side is an appropriate reference for estimating the radial head diameter by using 3D-based measurements and comparing right and left radial head diameters in healthy individuals. Second, we evaluated to what extent conventional 2D-based measurements in the standard axial CT slices are in agreement with 3D-based measurements.

Two morphological studies of Swieszkowski et al. [7] and Koslowsky et al. [17] investigated right-to-left differences of radial head diameters within respectively 8 and 18 cadaveric elbow pairs. They did not found significant differences. Contrary, our data demonstrated small but significantly larger diameters for the dominant right side. This difference in findings between our study and the latter studies may be explained by the difference in applied measurements techniques. As there is no consensus about the optimal selection reference in radial head arthroplasty, some surgeons prefer measuring the radial head diameter at, e.g., the articular facet [5]. We demonstrated also a larger diameter for the dominant right side at this level.

Two studies investigated methods for selecting the diameter of a radial head implant using anatomical landmarks of an ipsilateral bone, instead of using the contralateral radial head [5, 6]. Alalobi et al. [5] used the curvature of the lesser sigmoid notch in the proximal ulna as reference. They found that the reliability of this landmark to estimate radial head diameter was only moderate. Leclerc et al. [6] used the width from the lateral aspect of the capitulum to the lateral trochlear ridge in the distal humerus to estimate the radial head diameter. Measurements showed very strong correlations, based on a best-fitting line. In some individuals, however, the radial diameter deviated approximately 3 mm from this best-fitting line. In the current 3D CT study, we showed that right-to-left diameter differences as observed in single individuals were not larger than 0.9 mm, suggesting that the contralateral side is a better reference. Compared to the commonly available implant sizing increments, the anatomical right-to-left differences can be considered minimal, as it would not affect selecting implant size. The uninjured contralateral side can therefore be considered a suitable reference for selecting the implant diameter in radial head arthroplasty, without the need to correct for hand dominance.

Considering 2D-based diameter assessment, we showed that the radial head is more elliptical rather than perfectly circular, since the minimum and maximum diameters within radial heads differed significantly. This is consistent with previous findings, which showed that radial head is not always circular, but often oval-shaped [17–19]. Most radial head implants, however, are still circular, since elliptical implants would increase the technical difficulty in placing radial head implants although this may result in poor replication of the physiological kinematics of the radial head [14, 20]. Based on our results, if only the minimum diameter in an axial slice would be used as selection reference, the implant size could deviate up to 2.0 mm from the 3D-based diameter. This deviation is similar to the 2 mm diameter increments of implants, which may result in a difference between sizing up and sizing down. By averaging minimum and maximum diameters, the radial head diameter can be better approximated with a maximum difference of 1.0 mm compared to 3D-based diameter. The manual measurements of minimum and maximum diameters showed very high interobserver agreement, which is consistent with previous findings [4].

A limitation of a technique that uses CT images is lack of information of the articular cartilage because this is not visible. Therefore, our estimates could deviate slightly from true radial head size. Another important limitation of this study is that all participants were right-handed, which does not provide information about the right-to-left differences in left-handed individuals. Although not proven in this study, we do not expect larger right-to-left differences in left-handed individuals. In this study, we showed that our proposed 2D-based method for estimating the radial head diameter was comparable with the 3D-based method, and therefore suitable for clinical practice. However, future biomechanical and clinical studies are needed to evaluate to what extent the selected implant size actually restores the native elbow kinematics when using these measurement methods.

Besides choosing the appropriate implant diameter, the surgeon also has to restore bone length. The height of the implant relative to the surrounding bones can be altered intra-operatively by either removing additional native bone or by adjusting the collar size of the implant. Previous studies already assessed anatomical landmarks that guide the height of the radial head [21–24]. One of these studies concluded that measurements based on contralateral images of the healthy elbow were accurate in predicting radial head implant length to avoid over lengthening [21].

In conclusion, our study showed that the right radial head diameters is slightly larger in right dominant individuals. However, in radial head arthroplasty, this right-bias is not clinically relevant, as it would not affect choosing implant size based on the contralateral side. The uninjured contralateral side can therefore be considered a suitable reference for selecting the implant diameter. For standard clinical practice where 3D-based evaluation may not be available, we recommend using standard axial CT slices to measure the average of the minimum and maximum diameter, for estimating the radial head outer diameter.
